# Transforming through leadership: a qualitative study of successful American Indian Alaska Native behavioral health leaders

**DOI:** 10.1186/s12889-019-7600-9

**Published:** 2019-09-18

**Authors:** Brenda J. Freeman, Gary Bess, Candace M. Fleming, Douglas K. Novins

**Affiliations:** 1 Counseling and Educational Psychology, College of Education/Cooperative Extension, University of Nevada, Reno/0281, Raggio Building Rm 3007, Reno, NV 89557-0281 USA; 2 Gary Bess Associates, Paradise, CA USA; 3 Community and Behavioral Health, Centers for American Indian and Alaska Native Health, University of Colorado Anschutz Medical Campus, Aurora, CO USA; 4 Psychiatry and Community and Behavioral Health, Centers for American Indian and Alaska Native Health, University of Colorado Anschutz Medical Campus, Aurora, CO USA

**Keywords:** American Indian Alaska Natives, Human services, Leadership

## Abstract

**Background:**

Effective leadership is vital in the struggle to decrease the behavioral health disparities between the US population and American Indian Alaska Native (AIAN) communities. AIAN communities have a pre-colonization history of highly effective leadership, yet some AIAN leadership traditions have been eradicated through decades of trauma and genocidal efforts. There is a paucity of research on AIAN public health leadership, and most existing research relies on samples of individuals holding leadership positions rather than individuals purposely selected because of their effectiveness. The aim of the study was to investigate the experiences of successful AIAN behavioral health leaders and present an emerging AIAN public health leadership model.

**Methods:**

Thirty-eight public health leaders in the Substance Abuse and Mental Health Service Administration (SAMHSA) funded Circles of Care project were observed over the course of their three-year leadership role. Stringent criteria for successful community participatory leadership resulted in the selection of 11 of the 38 leaders for inclusion in the study. Ultimately eight leaders (21% of the population of observed leaders) participated in the study. Semi-structured, one-on-one qualitative interviews were conducted. The methods were informed by phenomenology and the data were analyzed using a thematic content analysis approach.

**Results:**

The analysis resulted in ten themes: Hopeful Vision for the People, Cultural Humility, Awareness of Historical Context, Purpose Driven Work Behavior, Cultural and Bi-Cultural Knowledge, Trusting a Broader Process, Caring Orientation, Holistic Supervision, Community Centered, and Influence Through Education. Respondents were strongly motivated by a desire to help future generations. They described their success in terms of the application of traditional AIAN values such as cultural humility and community orientation, but also relied heavily on task orientation. An emerging AIAN leadership model is presented.

**Conclusions:**

It is important to encourage AIAN public health leaders to employ leadership research and models conducted or developed in the context of AIAN communities. The emerging model presented in this study could serve as an initial basis for AIAN leadership training. Given the challenging context of AIAN leadership, the lessons taught by these successful leaders could be adapted for use by leaders in non AIAN settings.

## Background

Decreasing the behavioral health disparity between the US population and American Indian Alaska Native (AIAN) communities remains a significant public health challenge. AIAN populations have higher rates of trauma, posttraumatic stress disorder, depression [1], suicide [2], and substance abuse [3]. Failed government efforts to reduce disparities have led to a policy shift toward directly funding AIAN communities to deliver services designed specifically for each unique community [4].

Direct funding of AIAN communities to develop local solutions to public health problems creates an unprecedented opportunity for AIAN public health leaders to step forward, leaders with skills to facilitate community consensus, marshal systems-wide community change, and garner state and federal resources. Passed down from generation to generation and thriving within the fabric of AIAN culture is a millennia of leadership traditions, cultural expertise, and the fortitude required to confront the challenges of behavioral health disparities. Yet the compounded impact of over half a millennia of genocidal efforts, including introduced diseases, removal and relocation, massacres, boarding schools, forced religious conversion, and prohibition of cultural practices [5] has likely diminished the capacity of some AIAN communities to pass down culturally-embedded leadership traditions.

Literature on AIAN leadership in behavioral health is scant, but existing literature addresses the importance of blending western and AIAN approaches and underscores the reliance of AIAN leaders on a relational and horizontal, rather than a hierarchical, leadership stance [6]. The resiliency of AIAN leaders to persevere in a context of grueling challenges such as poverty, broken tribal government systems, and scarcity of resources is a distinguishing factor of AIAN leadership [7]. No published AIAN public health leadership model was found.

The goal of our study was to understand the attributes, meaning and strategies of effective leadership in AIAN communities. We aimed to gain this understanding by listening to the experiences, voices, perspectives, and teachings of the respondents. We hoped to contribute an emerging model of AIAN behavioral health leadership, one which could provide a basis for training and supporting leaders working to reduce health disparities in the unique cultural context of tribal communities. We conducted the study because there is scant research specific to AIAN behavioral health leadership and because the samples of available studies were drawn from individuals holding leadership roles without regard for actual success as leaders.

## Methods

### Design and approach

Qualitative approaches are often used in health sciences to explore and uncover understandings of complex societal phenomena, such as AIAN leadership, particularly when there is a dearth of research. Thematic analysis was applied to identify common themes across the core meanings of participants [8]. Phenomenology informed the study in that we sought to understand the every-day experiences of individuals and the ways in which they construct essential meanings [9]. To the extent possible the assumptions of the researchers were bracketed to focus on the meanings through the perspectives of the leaders themselves, though we also recognized the positionality and influence of the ethnicities of the researchers and the role of being AIAN technical assistance providers.

All four authors had over 20 years of experience working directly with tribal communities and working as behavioral health technical assistance providers for tribal communities. Two authors (one females and one male) were non-Natives with Ph.D. credentials. One author was a female, Native tribal member also with Ph.D. credentials. The fourth researcher, a non-Native male MD, served as senior author, providing guidance and consultation. The interviews were conducted by the three Ph.D. members of the team. Two of the interviewers worked in higher education and the third was a consultant. All were published authors in AIAN behavioral health, and all were experienced with qualitative research. The researchers had served as technical assistance providers for the participants, and had established professional relationships with all interviewees. The participants understood the reasons the research was being conducted and that the research team was biased toward providing support for AIAN communities.

### Participants

The research team decision to recruit participants from one initiative led to a smaller sample size, a weakness the we determined was offset by the advantages of a consistent leadership role across participants. Purposive sampling was used to select participants from the population of 38 project leaders across the first five rounds of the federal Substance Abuse and Mental Health Services Administration (SAMHSA) three-year program planning initiative, Circles of Care [10]. The initiative was established in 1998 with a primary goal of addressing public health disparities by designing community-based system of care service delivery models. Common leadership responsibilities for the project directors included establishing working advisory bodies, meting product deadlines, and engaging community members in a visioning process. In the first year of the three-year initiative, project leaders hired and supervised a small staff, usually comprised of one or more evaluators, a cultural specialist, a youth development specialist, a program administrative assistant, and a social marketing coordinator. In year one the leaders were required to use a Community Based Participatory Research (CBPR) approach to discover contextually relevant community strengths and health disparities across multiple service sectors. This effort was accomplished by initially conducting a community needs assessment and a multi-dimensional description of current assets and available services. During the second year leaders worked with stakeholder groups to translate the data into service delivery components. In year three leaders guided staff, partnership agencies, and community teams through a process of refining the service delivery components, conducting a feasibility study, adjusting the model, and seeking funding to implement the model.

Common leadership challenges included human resource issues (e.g. staff recruitment and turnover), building relationships with entrenched agencies, working across a multitude of AIAN cultures, and building partnerships with non-Native programs and regional, state, and tribal governments. Securing family member involvement and bridging the gap between the voices of families and the proclivities of service providers often proved challenging.

**Fig. 1:**
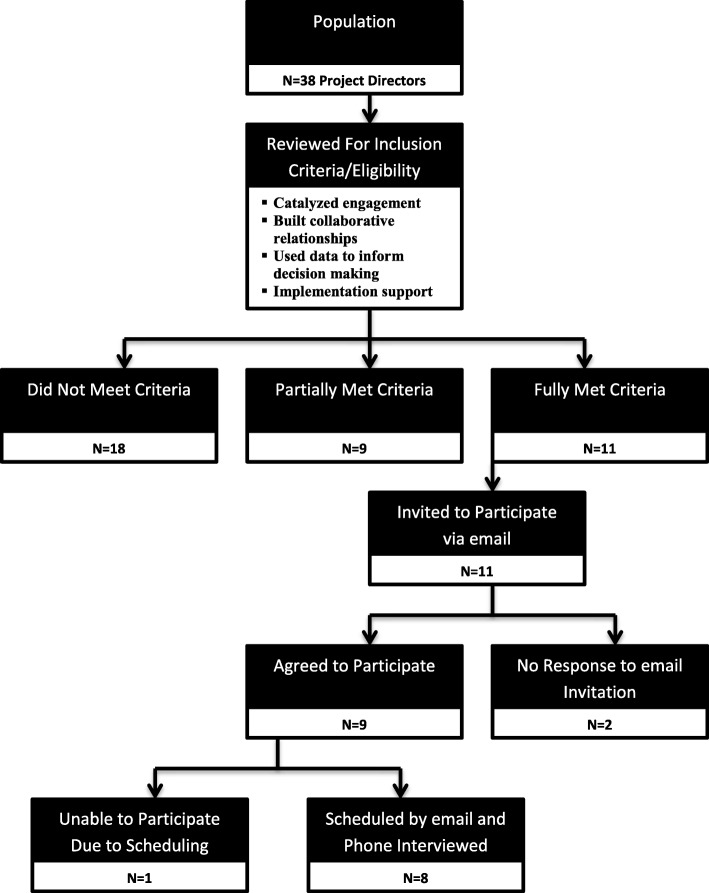
The number of potential participants in the population, the number of potential participants after the application of the inclusion criteria, and the process leading to the identification of the final group of individuals invited to participate in the study

All potential participants held the same leadership role (project director), leadership goal (system of care program development to reduce health disparities), broad approach (CBPR), and time constraints (3 years). The criteria used to operationally define successful leadership were: 1) the leader significantly catalyzed youth and family engagement; 2) the leader developed strong collaborative relationships across behavioral health and other service sectors; 3) the leader collected and effectively used local data to inform the development of a sound service delivery model, and 4) the leader secured support for partial or full implementation of the model. The determination of the eligibility was based upon our review of written products, live observation of each leader across 3 years within the context of their communities, monthly telephone consultations, and observation of interactions with staff and community members. As shown in Fig. 1, the participants agreed upon by all 4 research team members as having met the full criteria for the definition of successful AIAN leadership were included in the study (*n* = 11). A second list those rated as having partially met the criteria (*n* = 8) was compiled for use in the event that data saturation was not achieved with the first list. The 18 project directors who did not meet the selection criteria were removed from consideration. Many of those removed were effective leaders, but external circumstances led to an inability to meet the inclusion criteria. Examples of the circumstances were a short tenure in their role, debilitating staff turnover, political factors undermining the potential for success, lack of tribal permission to hire a full staff, lack of prior leadership experience, and tribal government misuse of project funds.

To protect the identities of the participants, only general demographics are shared. The participants included 1 male and 7 females from urban, rural, and reservation tribal communities from the southwest, northern plains, northwest coastal, California, and southeast tribes. Half the participants were mid-career, between the ages of 30 and 45, and half were late career, between 46 and 65. There were no participants under the age of 30 or over the age of 65. All the participants lived and worked within or near the boundaries of the communities they served. The degree of proficiency with Native language varied from limited language understanding to full proficiency. All participants had bachelor’s degrees; two had terminal degrees.

### Data collection

The 11 potential participants were contacted by e-mail and invited to participate in a semi-structured, hour-long interview without compensation. Two of the 11 did not respond to the invitation, and one responded but was unavailable to participate within the prescribed timeframe. The remaining eight participants consented to be interviewed, and were each assigned to a research team member who used email to schedule the phone interview. Participants gave oral informed consent at the beginning of a 45 to 120 min semi-structured telephone interview and were given the option of digital recording for transcription or note taking. The initial interview guide was designed by one team member. It was then reviewed individually by the other researchers. The final protocol, shown in Table 1, included eight broad stem questions and one to three sub-items to be used if needed to foster elaboration. Permission to conduct a second interview to clarify content was sought and received from all interviewees. No second interviews were conducted.

**Table 1:** Interview Protocol: Topics and Stem Questions

Topic	Stem Questions
Introduction	What does it take to become a strong leader in an American Indian Alaska Native community?
Goal Attainment	What, specifically, do you feel you have accomplished as a leader?
	Describe the process by which you engaged your team in planning and goal attainment?
Leadership approach and strategies	On a practical level, how did you approach the day-to-day leadership?
	How do you communicate your vision (goals) to others on your team?
	What leadership strategies do you use to help employees accomplish their work?
Leadership traits	I know it is sometimes awkward to talk about our personal positive traits, but will you please describe what it is about your personality, values, and/or beliefs that makes you an effective leader?
Leadership Development	Knowing that a leader puts a lot into the role and possibly changes along with the program, where were you at the beginning and how did you grow or change over the course of the program?
Leadership and AIAN Culture	What role did culture play in your accomplishments as a leader?
	Are there aspects of organizing and planning programs in Native communities that are unique and not transferable to other cultures and settings? If so, please describe.

### Data analysis

The data analysis was conducted free of theoretical constraints, but guided by two broad questions: “What does leadership mean to successful behavioral health leaders in AIAN communities?” “How do the participants understand the leadership characteristics and strategies used to bring about change in AIAN communities?” Iterative applied thematic analysis was conducted to discern emergent themes. Using NVivo 11 [11] one researcher coded the text for significant segments, attached theme tags, and established the codebook. The coding was segmented and adjusted by two other research team members. After data coding was completed, the research team worked together to examine the commonalities across the data, similarities and differences, and emergent themes, and the relationships between themes. The final theme selection was conducted through frequency analysis and research team consensus. Coded segments supporting the themes were identified in no fewer than six source interviews for all themes. Data saturation was achieved with the sample of 8 participants, fewer than the minimum standard for data saturation in thematic analysis. The researchers attributed this to the homogeneity of the purposive sample.

We synthesized the findings from the analysis to develop an overarching model of AIAN behavioral health leadership. The manuscript was returned to participants for member checking and was adjusted accordingly. To protect confidentiality participant numbers are not assigned to quotes in the results section.

## Results

The analysis resulted in ten themes: Hopeful Vision for the People, Cultural Humility, Awareness of Historical Context, Purpose Driven Work Behavior, Cultural and Bi-Cultural Knowledge, Trusting a Broader Process, Caring Orientation, Holistic Supervision, Community Centered, and Influence Through Education. The essence of the themes and frequencies are shown in Table 2.

**Table 2:** Coding, themes, and frequency of participant endorsement

Essence	Main Theme	Revelatory Theme	Frequency of Participants Coded for Theme
Foundational beliefs about leadership and the role of leaders in AIAN society undergird the work and motivate the leader. The work is difficult and fraught with challenges, yet the leaders persisted because of a foundational commitment to the people.	Beliefs about leadership in tribal communities	Hopeful Vision for the People	8
		Awareness of Historical Context	7
An inherent or learned disposition of humility was an essential trait across the successful leaders.	Leadership traits or dispositions	Cultural Humility	8
Relationally based leadership knowledge, skills, and strategies coalesced into a leadership approach that catalyzed community impact.	Leadership knowledge and strategies	Cultural and bi-cultural knowledge	7
		Purpose Driven Work Behavior	8
		Trusting a Broader Process	7
		Holistic Supervision	7
		Caring Orientation	8
		Influence Through Education	8
		Community Centered	8

### Hopeful vision for the people

Respondents reported being motivated to achieve their goals because of a deep sense of hopefulness and responsibility for *the people*, a term used by respondents interchangeably to refer to ancestors, elders, cultural group, tribe, or community. Respondents were passionate in their description of being motivated by a hopeful orientation toward others and a recognition that those who are capable to lead must accept the responsibility to lead. As stated by two participants:

*“In order to have that level of commitment you have to hold yourself responsible to the people you are trying to serve..... And there are so many levels to that. When you are making decisions for your program you are seeing the faces of the people of your community … it goes beyond your needs and wants. The words that in our language are [Native language phrase] and it means, ‘the ones who are hardy.’”*

*“[Native language phrase] is a phrase that means ‘standing for our people and advocating hope’ … it says that together we work to honor and empower our people.”*

Other participants voiced Hopeful Vision for the People as working on behalf of those who came before them and for future generations, wrapping their day-to-day work with broader meaning and purpose. Illustrations from the data include statements denoting the importance of living into the expectations of grandparents, a broad commitment to ancestors, and the dream of a better life for future generations of AIAN children. One respondent stated:

*“We are committed to future generations. My approach to work is not as a job or a project--it is to understand my role in [tribal] society. We aspire for a greater future for our people with sustained importance today and tomorrow.”*

### Cultural humility

Cultural humility encompasses the perspective that community expectations or spiritual forces rather than personal career ambition is the rationale for leadership in AIAN communities. Despite holding a leadership position with a supervisory title, participants were hesitant to self-identify as leaders, instead noting that good leaders are good followers and that all voices matter.

*“I remember seeing something that said people choose their leaders. I really believe that—that people choose who they want to lead them and I decided within myself I’ll continue to lead now and if they want me to play a background role I’m fine with that too--as long as I’m still making a difference... A good leader has the capability to be a good follower as well.”*

Consistent with a humble perspective participants described their leadership style as facilitative, transparent, flexible, and relational, avoiding terminology reminiscent of hierarchical leadership.

*“Allowing myself to have a free voice allows others to have a free voice … I mean if one is rigid and you think your voice is the only one that counts then obviously you are going to shut doors. For me I believe that everyone can contribute to a project no matter what it is... I allow freedom for myself; therefore I allow it for others. I truly believe that everyone comes to the table with something to give, and it has been the greatest thing for me to allow other to contribute in that way.”*

### Awareness of historical context

Participants described awareness of cultural history as a foundational trait from which leadership behavior arises. As stated by one participant, “We asked the staff, ‘what does it mean to be a Native American?’ We had to answer these questions ourselves.” Illustrations of the impact of historical context on leadership derived from the data set include recognition of multigenerational teachings, family expectations, intergenerational trauma, and traditional cultural roles.

*“ … I do presentations on historical trauma in our community and other places. I talk about boarding schools and the impact it has had on American Indian families. I share my own story--my mom went to boarding school. I was raised in an alcoholic family and was in and out of foster care. I think a lot of my own personal experiences make me really want to make sure that we are making changes.”*

### Purpose driven work behavior

Consistent across participants was a strong drive for achievement manifested as task orientation. As indicated by one participant, “I have a very driven personality, and that is not going to change.” All participants reported specific tasks they were driven to accomplish to reach their broader purpose, such as structured meetings to track progress toward goals, detailed task timelines, meticulous follow-up work, data based decision making, systematic tracking of funding requirements, and use of process evaluations to improve efficiency. Some participants balanced driven work behaviors with employee relationships, as indicated by this participant:

*“We met as a staff weekly and we had extended meetings to plan—two or three days, if necessary. We broke down the process into high detail. I think I’m open and supportive, but pretty direct with deadlines and the consequences that could happen if we don’t meet those deadlines.”*

### Cultural and bi-cultural knowledge

Knowledge of Western and AIAN culture were described by participants as foundational to their success. Examples of cultural knowledge were understandings gained from immersion within the culture, cultural practices, speaking the Native language, grappling with AIAN cultural identity, and understanding unwritten cultural norms unique to the community. Bi-cultural knowledge was as important as AIAN knowledge in that the leaders noted the crucial role of bridging the gap between AIAN and Western culture. Though some participants reported easily-accessible knowledge, such as being well informed on local behavioral health prevalence data, the majority of the responses reflected deeper understandings gained through involvement in both AIAN and Western worlds.

*“Being grounded in the culture allowed me to be culturally appropriate with the work. There is a Western and a cultural perspective … but don’t get me wrong—both types of systems exist. Having knowledge of traditional systems and Western systems allows me to integrate the Western systems in a beautiful way that allows the two worlds to align.”*

Respondent statements on the issue of AIAN potential leaders leaving their communities, perhaps leaving tribal land, to seek an education or other experiences in Western society only to return to their community as an outsider highlighted the strain of balancing Western and traditional knowledge and voice. As reported by one participant: “*I was trained in the western model and needed to ‘teach myself how to speak again.’ My new language was not conducive for my work in our community.”*

Awareness of taking care to speak the language of the audience to enhance stakeholder understanding was a skill reflected in all participant interviews. One participant described bridging the language of the community on evaluation and data collection.

*“I know how to speak to the community so they will understand what we are trying to convey to them without using the research terms, without saying ‘data’ or ‘analysis.’ I was a bridge between two worlds... When I needed to speak to the providers to sound more authentic (like I knew what I was doing) I could use those terms with them.”*

### Trusting a broader process

Interviewees identified the importance of trusting a broader, sometimes spiritual process, one that is outside the direct control of the leader. Stated by one participant, “In Native everything has a purpose and there are seasons and you don’t rush into things. You just go with it, because nature and the creator have dealt this path and you follow it.” This process was described as a deep belief in the assumption that if the process is trusted, the outcome is as it should be. As described by other participants: *“If it is put in front of you at that exact moment, then it is exactly where you need to be, exactly what you need to attend to … trust that it is all going to work out just fine.” “We learned that we can trust our community … and that trust becomes reciprocal … we trust their ideas and they trust our process.”*

The act of trusting the process was consistently associated with positive results. As one participant described: “*There’s times when you have to change priorities on a dime and you have to be okay with that. Trust that.”* Some participants described trusting the process as intentionally letting go or giving up control, making a conscious choice to abandon the plan in favor of the naturally evolving process.

*“I would plan, have an agenda--this is what we are going to talk about … this is what we are going to do … this is how long it is going to take us--but I found that meetings and who you had at the meetings took on a life of their own. You can’t be a control freak. You have to be willing to let the meeting flow in the way the meeting needs to flow. Sometimes we would hit on one topic, sometimes we would spend the whole meeting just talking about the first item on the agenda and sometimes we would shoot through the whole agenda and start talking about something totally different. What came up was important and it was something that had to be discussed or processed with the group. So I had to really realize that and tell myself to let go … let go of … a ‘this is what we have to do’ attitude.”*

### Caring orientation

Participants approached the work of leadership with a deep caring for others. One participant described the importance of caring about the next generation of young people: *“I try to be a role model … my focus is on young Native people, as the youth really need our mentorship.”* Another participant stated the importance of kindness towards others as part of their leadership style:

*“I try to come from a kind heart … one of the things I took from my grandfather. People came to him because he was kind with his heart and kind with his spirit. That’s what I have carried and that is why I’m able to [lead] … I don’t think my grandfather saw colors of people. He was just kind to them.”*

### Holistic Supervision

Holistic supervision involved the leader understanding the staff as individuals, listening to staff, understanding work-to-home balance, and viewing the individual holistically and from a strength-based perspective. As stated by one respondent:

*“Understanding what the employee is going through right now and how can you support them and adapt to their learning style is critical. You must adjust your management style to help them accomplish their tasks … . It’s helps them and you if you understand their whole situation and look at them holistically and not just as an employee.”*

Participants voiced the importance of understanding the entirety of the staff members’ situation beyond their work role, including their natural strengths, family situations, and their spiritual lives.

“*Especially in a tribal setting it’s very … what do you call it? It’s very circular. Just because they are at work doesn’t mean they turn off the issues they are having at home with their children, their spouse, or with their sick mother. It’s all encompassing …* ”

### Community centered

Community engagement was reported as a central objective and a key means to goal attainment. Participants responded to almost every query from the vantage point of community relationships. A word frequency analysis of the transcripts showed that the word, *community*, was by far the most often used term across the interviews. Participants reported that to bring about change, a community centered leadership approach based in building relationships across community entities was essential. As stated by one respondent:

*“Everyone was doing their own thing. It was hard to get other agencies and even some community members who maybe felt loyal to other agencies to be a part of this. We needed to get them to understand that this vision is for the whole community and this is something that is going to be shared with the whole community--it isn’t ours; it belongs to our community. That was challenging. It took a few years to get the community to work with us and be open to it. Now it’s just amazing. We partner. We subcontract with each other. We work on projects together. It just has changed so much.”*

Resistance to change, no matter whether from service providers or entrenched leadership, was addressed through relationship building, finding common ground, establishing reciprocal partnerships, and actively building trust.

### Influence through education

Though all participants reported the need to bring about community change, rather than utilizing direct persuasion participants described employing the indirect strategy of education (often awareness raising) within the context of community relationships. Building relationships and gently educating government officials, public agency staffs, tribal leaders, community members, and non-tribal service providers were reported as critical to goal attainment. Described by one participant:

*“You need to be prepared to tell the story over and over again. The more you educate about project and vision, the more people understand. It takes time for information to sink in … repetition is important. It took a lot longer to educate county and state government.”*

#### Emerging model

**Fig. 2:**
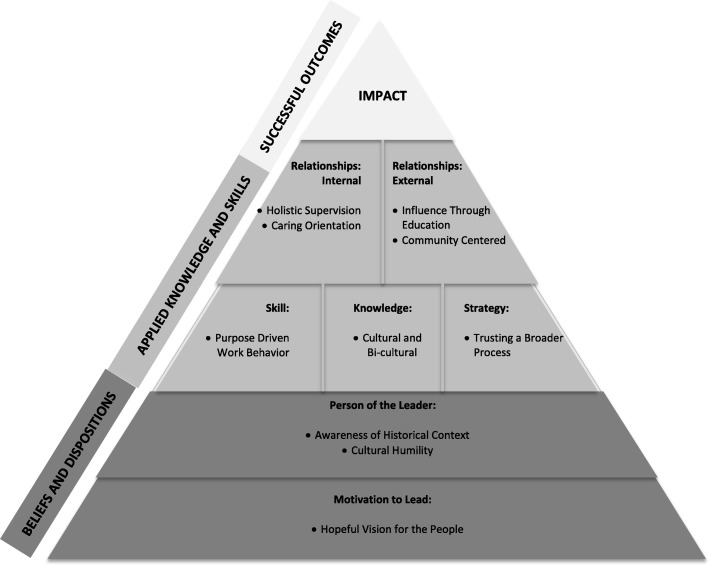
A visual depiction of the conceptual model of AIAN behavioral health leadership emerging from the analysis. The foundation of the model is comprised of AIAN cultural and historical leader understandings and beliefs

A conceptual model of AIAN behavioral health leadership emerging from the analysis is shown in Fig. 2.

The model was developed by the research team through considering the context of the themes, the relationships between the themes, and the degree to which participants emphasized each theme. The foundational level of the model is a motivation to lead that is deeply rooted in a hopeful vision for the people, referring to ancestors, the community, the culture, and future generations. Regardless of the interview question, participants repeatedly returned to this concept, implying that it fuels the resiliency necessary for leaders to persevere in the face of significant challenges. Building upon this foundation are two traits of the leader—*Awareness of Historical Context* and *Cultural Humility*. Though participant perspectives on culture varied from traditionalists to contemporary urban AIAN culture, the concept of culture, the lived-experience of culture, and the keen awareness of personal and cultural history were described as bedrock for effective leadership. *Cultural Humility* derives from a belief that the power to lead is transmitted to the individual from the spiritual world, not from the mere efforts of individuals. Described by Black Elk [12] (pg. 127), “if I thought that I was doing it myself … no power could come through. Then everything I could do would be foolish.” Knowledge, skills, and strategies emanate as natural byproducts of self-awareness and cultural humility. Effective AIAN leaders use a wealth of skills, knowledge, and strategies, but *Purpose Driven Work Behavior*, *Cultural and Bi-Cultural Knowledge*, and *Trusting a Broader Process* were three attributes emphasized across participant interviews. Built upon these themes are four relational skill sets, which serve as the conduit for bringing about change. *Holistic Supervision* and *Caring Orientation* were descriptive of the nature of relationships with employees. *Influence through Education* and *Community Centered* depicted the nature of relationships with external stakeholders. The operational principle at this level of the model is that knowledge, skills, and strategies are applied through a relational schema. As noted by one participant, even firing an employee is relational: *“In the real world when you don’t work on the reservation, if you fire someone you will never see them again, but on a reservation that isn’t true. When I fire someone … they won’t talk to me for the first couple times they see me, but eventually they will. I don’t sever that relationship … Leadership is very different in a tribal community.”* The apex of the model, *Impact*, was rarely elucidated by participants. Perhaps because of cultural humility, participants were reticent to discuss the impact or the accomplishments of their work. However, since the selection criteria for the sample included significant accomplishments toward impacting behavioral health disparities, the apex represents tangible, community-level impact.

## Discussion

The epoch of the federal governmental working *on behalf of tribes* to address behavioral health disparities has been supplanted by an era of the government *working with* tribal entities as AIAN communities discover local solutions to behavioral health disparities. Given the overwhelming adverse consequences of AIAN health disparities, strong leadership to overcome formidable barriers is essential. AIAN leaders such as the eight remarkable individuals interviewed in our study are rising to the challenge.

*Awareness of Historical Context* is triangulated with literature on the importance of understanding the context of intergenerational cultural trauma in AIAN communities [13]. Training in trauma-informed care in AIAN communities is common, but the supporting quotes for *Awareness of Historical Context* and *Cultural and Bi-Cultural Knowledge* suggest a deeper level of understanding, one that recognizes personal familial trauma, knowledge of cultural and tradition, and a keen awareness of the broader history of traumatic events against AIAN peoples. Two of the themes, *Holistic Supervision and Caring Orientation,* are triangulated with extant research findings that servant leadership is dominant in cultures that value low power distance [14]. Our participants led with a personal power based in compassion and kindness, rarely referencing positional power in the interviews. In addition to reflecting servant leadership, *Holistic Supervision* and *Caring Orientation* may be an extension of *Awareness of Historical Context*. It is possible that the deep knowledge of cultural trauma reflected by our participants leads to a self-assigned leader responsibility to be an agent of healing of trauma for staff and other community members, or at least to take care not to partake in horizontal trauma of others in the community. In some work environments this level of compassion for employees could be construed as a lack of boundaries between supervisors and staff. In the context of AIAN behavioral health leadership, rather than reflecting a boundary problem our analysis suggests that participants intentionally operated with a low power distance from employees (consistent with the theme, *Cultural Humility*). Participant comments reflected skill at finding a balance between task orientation and caring, expecting employees to meet purposeful work goals while simultaneously expressing compassion for personal trauma of employees. *Purpose Driven Work Behavior* reveals a task orientation not found in prior AIAN leadership research. Perhaps the relational orientation often associated with AIAN leadership masks the existence of an underlying achievement-driven, task orientation. Balancing a relational orientation with a task orientation echoes the ancient AIAN medicine wheel, where balancing between the quadrants is thought by some tribal cultures to be critical for health and spiritual wellbeing across the lifespan [15]. Consistent with the AIAN Community Readiness Model [16] and motivational interviewing [17]. *Influence Through Education* is one of the strategies considered ideal in communities where the readiness to change is marginal. This is consistent with the less directive approach to internal leadership that our interviewees described and perhaps helps them maintain a consistency in interpersonal engagement within and outside the team. Educating others to raise awareness rather than using direct persuasion may also reflect the high value our participants placed on respect for others. Taken together the themes, *Community Centered* and *Hopeful Vision for the People,* suggest that our participants found deep meaning in their work and were consequently strongly motivated by the hope of restoring strength and wholeness in their communities. Consistent with an Existential perspective [17, 18], meaning in work for our participants was reflected by commitment (emotional connection to the work), control (belief that one can impact a positive outcome), and challenge (capacity to embrace change).

The opening question of the interview protocol, intended as a transition into the interview, asked the participants their perspectives about the characteristics of *strong* AIAN leaders, whereas the remainder of the questions related to *effective* leadership. A review of the findings showed that the participants considered the concepts to be equivalent. Another interesting consideration is the demographics of the leaders, with most being middle aged and older females. An exploration of the interview with the male participant showed a higher level of task orientation and a lower support for the relationship themes than female participants. It is conceivable that the leadership model is in effect a model of effective female leaders (or elders). Matriarchal leadership, embedded in the history of some tribes, is thought to be relationally based [19] yet authoritarian leadership has been found to lead to higher productivity [20]. Paradoxically, participants in the study were highly productive, despite having both a high task oriented and a high relational orientation. More research is needed to understand this incongruency with the literature. When viewed through the lens of mainstream leadership research, *Trusting the Process* is a strategy that would likely be associated with either empowerment leadership or laissez-faire leadership [21].

The narrow scope of the study to community-based behavioral health planning is both a strength of the study and a limitation. Second, the theme *Community Centered*, may have been more a product of the SAMHSA expectations for grantees rather than an orientation toward leadership. Though the sample size was appropriate for an initial, exploratory study, the small number of participants may affect the applicability of the findings. The participants represented both urban and rural communities, yet the data were analyzed as a unified whole.

## Conclusions

The achievements of the study participants suggest that even in challenging conditions there are local leaders well prepared to address the myriad of health disparity challenges in AIAN communities. The eight participants achieved noteworthy behavioral health outcomes within a relatively short timeline while working in contexts with substantial barriers and complex cultural issues. The traits and strategies of these role models create the footprint for stronger AIAN public health leadership selection and training, particularly within the context of community based participatory leadership. The AIAN Behavioral Health Leadership Model (Fig. 2) is intended to make a contribution to leadership training programs for AIAN behavioral health leaders. The Beliefs and Dispositions described in the model, if confirmed and adjusted with further research, could inform selection of individuals for intensive AIAN leadership training as well as selection for behavioral health leadership positions. Some of the Applied Knowledge and Skills (e.g. Cultural and Bi-cultural Knowledge) could be directly taught, while others (e.g. Trusting the Process) might be expressed through story telling, a culturally relevant training approach in AIAN communities. Further research is needed to determine if the model translates to male leaders in AIAN communities, as well as to understand if the model is applicable to a broader range of human services and public health AIAN roles beyond CBPR planning initiatives.

## Data Availability

The datasets generated and/or analyzed during the current study are not publicly available due to the protection of individual privacy of participants, but may be made available from the corresponding author on reasonable request.

## Abbreviations

AIAN: American Indian Alaska Native
SAMHSA: Substance Abuse and Mental Health Service Administration
